# Proteomic study on neurite responses to oxidative stress: search for differentially expressed proteins in isolated neurites of N1E-115 cells

**DOI:** 10.3164/jcbn.18-31

**Published:** 2018-08-11

**Authors:** Koji Fukui, Shunsuke Okihiro, Yuuka Ohfuchi, Minae Hashimoto, Yugo Kato, Naoki Yoshida, Kaho Mochizuki, Hiroki Tsumoto, Yuri Miura

**Affiliations:** 1 Molecular Cell Biology Laboratory, Department of Systems Engineering and Science, Graduate School of Engineering and Science, Shibaura Institute of Technology, 307 Fukasaku, Minuma-ku, Saitama 337-8570, Japan; 2 Molecular Cell Biology Laboratory, Department of Bioscience and Engineering, College of Systems Engineering and Sciences, Shibaura Institute of Technology; 3 Research Team for Mechanism of Aging, Tokyo Metropolitan Institute of Gerontology, 35-2 Sakaecho, Itabashi-ku, Tokyo 173-0015, Japan

**Keywords:** oxidative stress, neurite, proteomics, aging, Alzheimer’s disease

## Abstract

Reactive oxygen species attack several living organs and induce cell death. Previously, we found axonal/dendrite degeneration before the induction of cell death in hydrogen peroxide-treated neuroblastoma: N1E-115 cells and primary neurons. This phenomenon may be connected with membrane oxidation, microtubule destabilization and disruption of intracellular calcium homeostasis. However, its detailed mechanisms are not fully understood. Here, we identified proteins after treatment with hydrogen peroxide using isolated neurites by liquid chromatography-matrix-assisted laser desorption/ionization-time of flight/time of flight analysis. Twenty-one proteins were increased after treatment with hydrogen peroxide. Specifically, 5 proteins which were secretogranin-1, heat shock protein family D member 1, Brain acid soluble protein 1, heat shock 70-kDa protein 5 and superoxide dismutase 1, were identified of all experiments and increased in isolated neurites of hydrogen peroxide-treated cells compared to the controls. Furthermore, secretogranin-1 and heat shock protein family D member 1 protein expressions were significantly increased in normal aged and Alzheimer’s transgenic mice brains. These results indicate that secretogranin-1 and heat shock protein family D member 1 might contribute to reactive oxygen species-induced neurite degeneration. Both proteins have been related to neurodegenerative disorders, so their study may shed light on neurite dysfunction.

## Introduction

Reactive oxygen species (ROS) attack living tissues and induce cell death.^(1,2)^ To prevent ROS-induced cell death, organisms have antioxidant defense systems such as antioxidant enzymes [superoxide dismutase (SOD), catalase (CAT), glutathione peroxidase (GPx), etc.] and compounds (vitamins, polyphenols, etc.). However, these defense systems gradually attenuate with senescence. Accumulations of age-related oxidative damages derived from the collapse of redox balance increase the risks of development and progression of severe diseases such as Alzheimer’s (AD), Parkinson’s, Huntington’s, and other neurodegenerative disorders.^(3,4)^ Based on the free radical theory of aging hypothesized by Harman in 1956,^(5)^ the relationship between age-related oxidative damage and brain dysfunction has been a subject of much study. The brain is particularly vulnerable to ROS, given that its ratio of oxygen consumption is about 20%.^(6)^ Polyunsaturated fatty acids, such as docosahexaenoic acid and eicosapentaenoic acid, are present in brain cell membranes and are easily oxidized compared to other fatty acids.^(7)^ If neurons receive sufficient damage from ROS, it induces cell death. The alteration and dissociation of neuronal networks by cell death, is difficult to recover. Therefore, to prevent ROS-related neuronal cell death, it is important to find early signs of neuronal changes prior to cell death induction.

Previously, we found that treatment with a low concentration of hydrogen peroxide induced axonal and dendrite degeneration.^(8)^ The neurites showed abnormal morphologies, such as fragmentation, shrinkage, and beading, a well-known marker of axonal degeneration. The number of dendrites significantly decreased in hydrogen peroxide-treated primary granule cells.^(9)^ Axons and dendrites play a crucial role in neurotransmission in the central nervous system, and several lines of evidence demonstrate a relationship between axonal degeneration and neurodegenerative disorders.^(10)^ However, axons and dendrites can contract and expand easily, and the detailed mechanism of neurite degeneration has not yet been elucidated. For these reasons, we are focusing on neurite functions. To clarify the mechanism of hydrogen peroxide-induced neurite degeneration in neuronal cells, changes in cytoskeletal and cytoskeletal-related protein expressions have been measured. The ratios of cleaved and phosphorylated forms of collapsing response mediator protein (CRMP)-2, which is a microtubule-related protein, are significantly enhanced in hydrogen peroxide-treated neurons.^(8,11,12)^ The ratio of microtubule-associated protein-light chain 3 II (MAP-LC3 II), which is marker of autophagy, and the levels of lipid hydroperoxide significantly increase in hydrogen peroxide-treated cells.^(8)^ These changes have also been detected in the hippocampal CA1 region of vitamin E-deficient and normal aged mice brains.^(13)^ Vitamin E is a natural lipophilic substances that acts as an antioxidant, and vitamin E-deficient mice have accelerated oxidative damage in every tissue. These results indicate that ROS-induced microtubule alterations, including membrane oxidations, may contribute to neurite degeneration. However, the detailed mechanism is still not understood. Furthermore, these previous experiments have relied on whole-cell lysates. In the present study, we isolated elongated neurites and identified various proteins in hydrogen peroxide-treated neurons.

## Methods

### Cell culture, animals and reagents

**Fig. 1:**
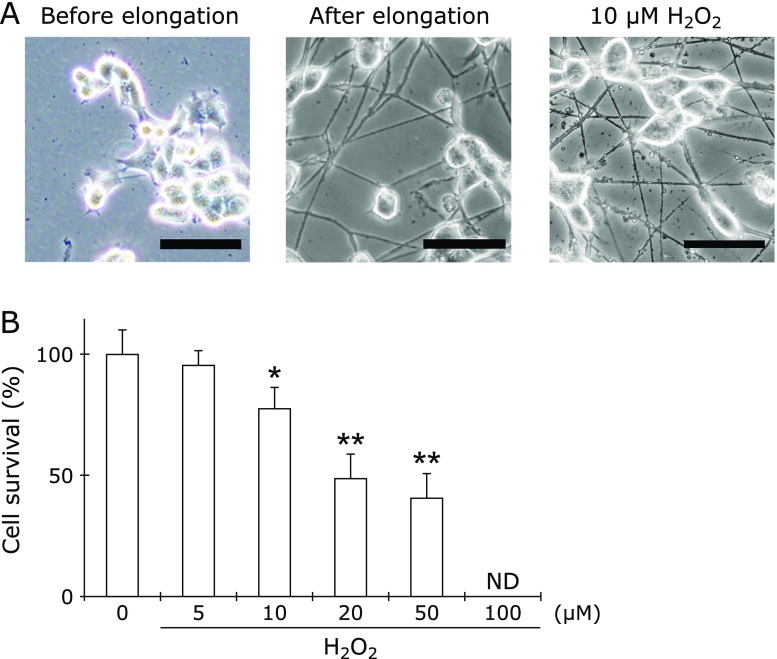
Morphology of N1E-115 cells before and after elongation of neurites, and induction of neurite degeneration in 10 µM hydrogen peroxide-treated N1E-115 cells (A). Scale bar is 50 µm. Hydrogen peroxide induces cell death in a concentration-dependent manner in N1E-115 cells (B). After treatment with various concentrations of hydrogen peroxide, dead cells were counted by trypan blue dye exclusion. Survival on untreated group was set to 100%. Data were analyzed by Dunnet’s test; * indicates *p*<0.05, ** indicates *p*<0.01. Each result represents at least four independent experiments.

N1E-115 cells (ECACC #88112303), which are derived from a mouse neuroblastoma C1300 tumor, were originally obtained from DS Pharma Biomedical Co., Ltd. (Osaka, Japan). Cells were grown in Dulbecco’s minimum essential medium containing 10% heat-inactivated fetal calf serum (FCS) (Biological Industries, Beit Haemek, Israel), 2 mM glutamine, 50 U/ml penicillin and 50 µg/ml streptomycin, and were plated in wells, dishes, or cell culture inserts of laminin [derived from mouse Engelbreth-Holm-Swarm (EHS) sarcoma]-coated plates at a density of 4.0 × 10^5^ cell/ml. Medium from cells cultured for 48 h was collected and used as conditioned medium. To elicit neurite elongation, after confirming cell adhesion, the medium was changed to conditioned medium containing 1% dimethyl sulfoxide (DMSO) solution. After 48 h, cells were used in experiments (Fig. 1A).

All animal experiments were performed with the approval of the Animal Protection and Ethics Committee of the Shibaura Institute of Technology. Three-month-old control animals (C57BL/6, male) were purchased from Japan SLC, Inc. (Hamamatsu, Japan). Twenty-four-month-old (C57BL/6, male) mice were obtained from the Tokyo Metropolitan Institute of Gerontology (Tokyo, Japan). AD transgenic mice [#008730, MMRRC034848, B6.Cg-Tg(APPSwFlLon,PSEN1*M146L*L286V)6799Vas/Mmjax, Alias/5XFAD] were purchased from the Jackson Laboratory (Bar Harbor, ME). All mice were maintained under controlled conditions of temperature (22 ± 2°C), 12 h light/dark cycle, and had free access to food and water. The aged-mice were acclimated to their new environment for one week before each experiment. Normal diet pellets (Labo MR Stock) were purchased from Nosan Corp (Kanagawa, Japan). After dissection, the cortex regions were homogenized and used as brain samples.

All other chemical agents were obtained from either FUJIFILM Wako Pure Chemical Co. (Osaka, Japan) or Sigma-Aldrich Co. (St. Louis, MO). All tissue culture plates and dishes were purchased from Becton Dickinson and Company (Franklin Lakes, NJ).

### Optimization of hydrogen peroxide concentration

 To determine the optimal hydrogen peroxide concentration against N1E-115 cells, cell survival was assessed using the trypan blue dye exclusion assay. After confirming neurite elongation, cells were treated with various hydrogen peroxide concentrations (0, 5, 10, 20, 50, and 100 µM) for 24 h, 0.8% (w/v) trypan blue in phosphate-buffered saline (PBS) was added to each sample, and the plate was incubated for 20 min in a CO_2_ incubator at 37°C. Cells were then washed with PBS at least three times. Photomicrographs of the cells were taken using an Olympus IX81 phase-contrast microscope (Olympus Corp., Tokyo, Japan) equipped with a DP71 digital camera (Olympus Corp.), after which the images were stored and processed on a personal computer. Photomicrographs used for analysis were selected randomly for each treatment and incubation condition. The number of living cells per unit of area was counted, and the data were presented as a percentage of the total number of cells. At least four wells were analyzed for all experimental conditions, and each experiment was repeated three times.

### Immunocytochemical analysis

To clarify the separation between cell bodies and neurites across insert membranes, we performed immunostaining. After 24 h of treatment with 10 µM hydrogen peroxide, N1E-115 cells were washed and scraped on the upper side of the insert, including cell bodies by a sterilized cotton swab, and the under layer of the insert was stained with β-actin and Hoechst 33258 dye. The cells or neurites were fixed with 4% paraformaldehyde (PFA) in PBS for 15 min at 4°C. Cells were then blocked with 10% goat serum in PBS for 1 h at room temperature (RT) and probed with mouse anti-beta actin monoclonal antibody (#ab8224, Abcam plc., Cambridge, UK) diluted 1:400 in PBS containing 1% goat serum, 1% bovine serum albumin (BSA), and 0.05% Triton X-100 overnight at 4°C. The cells were then incubated with Alexa Fluor 488-conjugated anti-mouse or anti-rabbit IgG secondary antibodies (Life Technologies Japan Corp., Tokyo, Japan) diluted 1:500 in PBS for 1 h at RT. Finally, to observe the status of nuclei, cells were stained with 1 µM Hoechst 33258. Photomicrographs of neurites were taken on a phase-contrast microscope equipped with a digital camera, stored, and then processed on a personal computer. Fluorescently labeled cells were observed and photographed with a fluorescence microscope. Immunocytochemical experiments were performed at least three times.

### Western blotting

All samples were homogenized in PBS and used in western blotting as described previously,^(9)^ with some modifications. Sample lysates were centrifuged and protein contents were determined using the Bradford assay (Bio-Rad protein assay, #500-0006JA, Bio-Rad Laboratories, Inc., Hercules, CA) according to the manufacturer’s protocol. Protein extracts (isolated neurite and brain homogenate samples of 10 and 30 µg, respectively) were separated on 10, 12 and 15% sodium dodecyl sulfate (SDS)-polyacrylamide gels and transferred to polyvinyliden difluoride (PVDF) transfer membranes (Immobilon; Merck KGaA, Darmstadt, Germany). The PVDF membranes were washed and incubated in blocking solution [Tris-HCl-buffered saline, pH 7.6 (TBS), containing 0.1% Tween 20 and 2% non-fat skim milk] for 1 h at RT. The membranes were washed in TBS containing 0.1% Tween 20, and then treated with each primary antibody {rabbit monoclonal anti-histone H1.0 antibody (EPR6536), 1:1,000, #ab134914, Abcam plc.; mouse anti-beta actin monoclonal antibody, 1:2,000, #ab8226, Abcam plc.; rabbit anti-chromogranin B polyclonal antibody, biotin conjugated, 1:500, #bs-0543R, Bioss Antibodies Inc., Woburn, MA, (Alias/Secretogranin-1); rabbit anti-heat shock protein family D (Hsp60) member 1 (HSPD1) (C-term) antibody, 1:1,000, #AP2895b-ev, Abgent Inc., San Diego, CA, [Alias/60 kDa heat shock protein (HSP)]; rabbit anti-heat shock 70-kDa protein 5 (Hspa5) polyclonal antibody, 1:10,000, #GTX127934, GeneTex, Inc. Irvine, CA (Alias/78 kDa glucose-regulated protein); rabbit anti-brain acid soluble protein (BASP) 1/Nap22 polyclonal antibody, 1:500, #bs-8662R, Bioss Antibodies Inc.; rabbit polyclonal anti-prefoldin 1 (E35) antibody, 1:500, #BS2245, Bioworld Technology, Inc., Louis Park, MN; rabbit anti-SOD1 polyclonal antibody, 1:500, #bs-10216R, Bioss Antibodies Inc.} overnight at 4°C. Anti-mouse or -rabbit IgG HRP antibody (Promega Corp., Madison, WI) was used as a secondary antibody at 1:4,000 dilution for 1 h at RT. All western blotting experiments were performed at least three times. All chemiluminescent signals were generated by incubation with the detection reagents (Immobilon; Merck KGaA, Darmstadt, Germany) according to the manufacturer’s protocol. For normalization of the bands for each protein, the membranes were reprobed with anti-α-tubulin rabbit monoclonal antibody (#2125, Cell Signaling Technology Inc., Danvers, MA). The relative intensities were determined using LAS-3000 (FUJIFILM Corp., Tokyo, Japan). Expression ratios were calculated by dividing each protein value by those of the α-tubulin forms using Image J software (National Institutes of Health, Bethesda, MD).

### Extraction of neurite proteins for iTRAQ labeling

Neurites isolated from cell culture inserts in the presence or absence of hydrogen peroxide were centrifuged at 15,000 rpm for 10 min at 4°C, respectively. After discarding supernatants, extraction buffer (7 M urea, 2% CHAPS, 50 mM HEPES, pH 8.5) was added and the samples were sonicated on ice. After adjustment of pH (from 8.4 to 9.0 by 1 M Tris-HCl sol.), the samples were incubated for 15 min at RT. SDS (20%), deionized water was added, and the samples were incubated for 10 min at 70°C. The samples were centrifuged at 15,000 rpm for 20 min at 4°C and resuspended in ice cold acetone. After incubation for 60 min at 4°C, the samples were centrifuged at 15,000 rpm for 20 min at 4°C. The pellet samples were resuspended in acetone and then centrifuged at 15,000 rpm for 20 min at 4°C. After discarding supernatants, the samples were dried and were resuspended in 0.2% SDS in 0.5 M triethylammonium bicarbonate (TEAB) buffer. Finally, protein concentrations were measured by the Bradford assay, and the samples were diluted to 1 µg/µl by 0.2% SDS in 0.5 M TEAB buffer. These were used as samples for the iTRAQ labeling.

### iTRAQ labeling

Extracted neurite proteins in the presence or absence of hydrogen peroxide were labeled with iTRAQ reagent using the iTRAQ Reagent 3-Assay Duplex Trial Kit (AB SCIEX, Framingham, MA) according to the manufacturer’s protocol with some modifications.^(14)^ Twenty µg of extracted neurite proteins were reduced by 4 mM tris-(2-carboxyethyl)phosphine (TCEP) for 1 h at 60°C, and were alkylated by 8 mM methyl methanethiosulfonate (MMTS) for 10 min at RT. The samples were trypsinized overnight at 37°C. iTRAQ reagents 114 and 117 were dissolved 70 µl ethanol and mixed with each sample. After mixing with two iTRAQ-labelled samples, mixtures were concentrated by evaporation, and strong cation exchange (SCX) load buffer (10 mM KH_2_PO_4_ in 25% acetonitrile (MeCN), pH 3.0) was added. Finally, phosphoric acid was added, the pH confirmed as 2.5 to 3.3, and the peptides were fractionated into seven fraction on Mono-Spin^TM^ SCX spin column (GL Sciences Inc., Tokyo, Japan) using 10 mM KH_2_PO_4_ in 25% MeCN containing 10, 25, 50, 100, 175, 350 and 1,000 mM KCl, pH 3.0. After evaporation and reconstitution in 2% MeCN/0.1% trifluoroacetic acid (TFA), the samples were desalted by a Mono-Spin^TM^ C18 spin column (GL Sciences). Eluates were evaporated *in vacuo* to dryness, reconstituted in 2% MeCN/0.1% TFA, and were subjected to liquid chromatography-matrix-assisted laser desorption/ionization-time of flight/time of flight (LC-MALDI-TOF/TOF) analysis.

### LC-MALDI-TOF/TOF

iTRAQ-labeled samples were separated and automatically spotted onto the MALDI plate using the direct nanoLC and MALDI fraction system (DiNa-MaP, KYA Technologies, Tokyo, Japan). Solvent A was 2% MeCN/0.1% TFA and solvent B was 70% MeCN/0.1% TFA. Four mg/ml α-cyano-4-hydroxycinnamic acid (CHCA) and 0.08 mg/ml diammonium hydrogen citrate in 70% MeCN/0.1% TFA was used as a matrix solution. After the samples were loaded onto a trap column (HiQ sil C18W-3, 0.5 × 1 mm; KYA Technologies Corp., Tokyo Japan), the value was switched and the peptides were separated with an analytical column (HiQ sil C18W-3, 0.1 × 50 mm; KYA Technologies Corp.) at a flow rate of 300 nl/min. The liquid chromatography (LC) gradient was as follows: 0–2 min, 0–5% solvent B; 2–60 min, 5–45% solvent B; 60–75 min, 45–100% solvent B; 75–85 min, 100% solvent B; 85–100 min, 0% solvent B. The flow rate of the matrix solution was 1,400 nl/min. LC eluent was mixed with matrix solution and spotted onto a MALDI plate at 30-s intervals between 5–90 min for a total 171 spots. An AB SCIEX TOF/TOF 5800 system and TOF/TOF Series Explorer software ver. 4.1 (AB SCIEX, Framingham, MA) were used for analysis of iTRAQ-labelled samples. For each spot, MS spectra were acquired in the positive ion mode between *m/z* 800 and 4,000 and accumulated from 400 laser shots in a randomized raster. MS/MS spectra were acquired using the following parameters and methods: collision energy, 1 kV; CID control, ON; laser shots, <4,000; minimum S/N filter, 50; minimum mass, 800 Da: maximum mass, 4,000 Da; acquision order/fraction, strongest precursors first; maximum precursors/fraction, 100; precursor mass window, 200 resolution (FWHM); metastable suppression, ON.

### Protein identification

All MS/MS data were analyzed by ProteinPilot software ver. 4.5 beta (AB SCIEX). Detailed analytical conditions were as follows: sample type, identification; cysteine alkylation, MMTS; digestion, trypsin; instrument, 5800; species, *Mus musculus*; ID focus, biological modifications; database, uniprot_sprot_can+iso_20100622+Contams+. Protein identification was based on the following selection criteria protein score (ProtScore) >1.3 (unused, *p*<0.05, 95% confidence) and at least one peptides with an ion score above 95% confidence. The ratios of each identified protein analyzed by LC-MALDI-TOF/TOF were calculated as the average ratio of 117/114.

### Genotyping of AD transgenic mice

Ends of tails were cut and DNA was isolated by a commercial kit using the manufacturer’s protocol (KAPA Mouse Genotyping Kit, #KK7153, NIPPON Genetics Co., Ltd., Tokyo, Japan). Transgenic amyloid precursor protein (TgAPP) and transgenic presenilin 1 (TgPSEN1) primers were designed by the Jackson Laboratory. The TgAPP primers were designed to amplify a 377 base-pair (bp) region; the forward primer was 5'-AGGACTGACCACTCGACCAG-3' and the reverse primer was 5'-CGGGGGTCTAGTTCTGCAT-3'. The TgPSEN1 primers were designed to amplify a 608 bp region: forward, 5'-AATAGAGAACGGCAGGAGCA-3', reverse, 5'-GCCATGAGGGCACTAATCAT-3'. Interleukin (IL)-2 was used as an internal standard for the RT-PCR experiments. The primer set for IL-2 was designed to amplify a 324 bp region: forward, 5'-CTAGGCCACAGAATTGAAAGATCT-3', reverse, 5'-GTA GGTGGAAATTCTAGCATCATCC-3'. PCR (T100 Thermal Cycler, Bio-Rad Laboratories, Inc.) was carried out with the following protocol: 15 s at 94°C, 15 s at 56°C and 15 s at 72°C. All amplifications protocols ended with a 10-min extension at 72°C. PCR cycle numbers for each gene were 35. After RT-PCR, all products were evaluated using 1% agarose gel electrophoresis.

### Statistical analysis

Data were plotted as means ± SE, and were analyzed by Dunnett’s test, with *p*<0.05 considered significant.

## Results

### Optimization of hydrogen peroxide concentration

Before identification of proteins in isolated neurites of hydrogen peroxide-treated N1E-115 cells, we optimized the hydrogen peroxide concentration. As shown in Fig. 1A, treatment with 10 µM hydrogen peroxide induced neurite degeneration such as beadings. Treatment with hydrogen peroxide induced cell death in a concentration-dependent manner in N1E-115 cells (Fig. 1B). At more than 10 µM of hydrogen peroxide, the ratio of cell death significantly increased compared to the control. Because large parts of cells did not remain on the surface of the culture dish, we could not count cell death in 100 µM treated samples. Since we wanted to observe changes in neurite-specific proteins prior to the induction of cell death, 10 µM hydrogen peroxide was used for further experiments.

### Isolation of neurites from a cell culture insert system

**Fig. 2:**
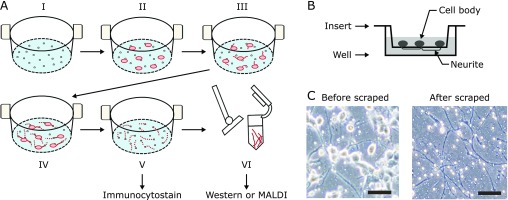
Schematic images of neurite isolation by cell culture insert system (A, B). Images before and after removal of cell bodies (C). Scale bar 50 µm. Small white dots are pores of cell culture membranes.

**Fig. 3:**
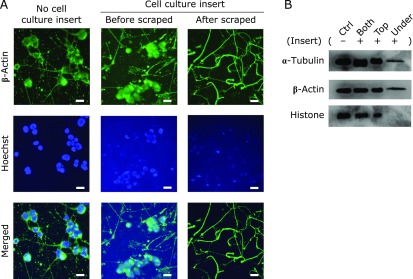
Confirmation of neurite isolation by immunostaining (A) and western blotting (B) before and after scraping the nuclei of N1E-115 cells from cell culture inserts. The cells were cultured on the insert, and then the neurites were extended to the reverse side of the insert through the membrane pores. The surfaces on the reverse side of the insert were scraped by sterilized cotton swabs and used as samples for western blotting. Each result is representative of three independent experiments. Scale bar 20 µm.

To isolate cell bodies and elongated neurites in hydrogen peroxide-treated N1E-115 cells, we used a cell culture insert system containing many small holes with diameters of 8 µm (Fig. 2A-I). Elongated neurites can pass through the holes and stick to the opposite surface of the insert membranes (Fig. 2A-IV and 2B). After neurite elongation, we scraped cell bodies from cell culture inserts by sterilized cotton swabs (Fig. 2C) and turned over the insert membranes for staining (Fig. 2A-V). We could not find histone proteins on the bottom surface (Fig. 3A). On the other hand, nuclear proteins were clearly stained on the top surface of the membranes before scraping. In western blotting assays, we could not detect nuclear protein in isolated neurite samples (Fig. 3B). We used this isolation method for further experiments.

### Identification of proteins in isolated neurites of hydrogen peroxide-treated N1E-115 cells by LC-MALDI-TOF/TOF

**Fig. 4:**
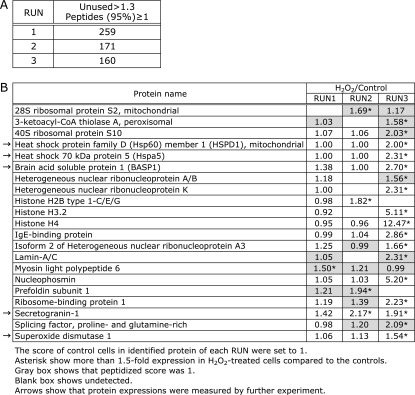
iTRAQ-labelled peptide samples from isolated neurites were analyzed by LC-MALDI-TOF/TOF. Total number of identified proteins of each RUN (A). Proteins with increased expression in isolated neurites of hydrogen peroxide-treated N1E-115 cells using LC-MALDI-TOF/TOF (B). The score of control cells in identified protein of each RUN was set to 1. Asterisks show more than 1.5-fold expression in hydrogen peroxide-treated cells compared to controls. Gray box shows that peptides score is less than 1. Blank box shows undetected. Other boxes show that the peptide score is more than 1. Arrows show that protein expressions were verified by further experiment. Each result (RUN) represents three independent experiments.

iTRAQ-labeled peptides samples were analyzed by LC-MALDI-TOF/TOF. Around 200 proteins were identified in each independent experiment (Fig. 4A). Detailed data of protein identification were summarized in Supplemental Table 1*. Figure 4B shows twenty-one proteins detected in at least twice experiments and with an increased expression ratio by hydrogen peroxide treatment. Five proteins represented by arrows in Fig. 4B were selected, and their alterations of expressions were confirmed in isolated neurites treated with hydrogen peroxide and in animal homogenate in subsequent experiments.

### Validation by western blot analysis

**Fig. 5:**
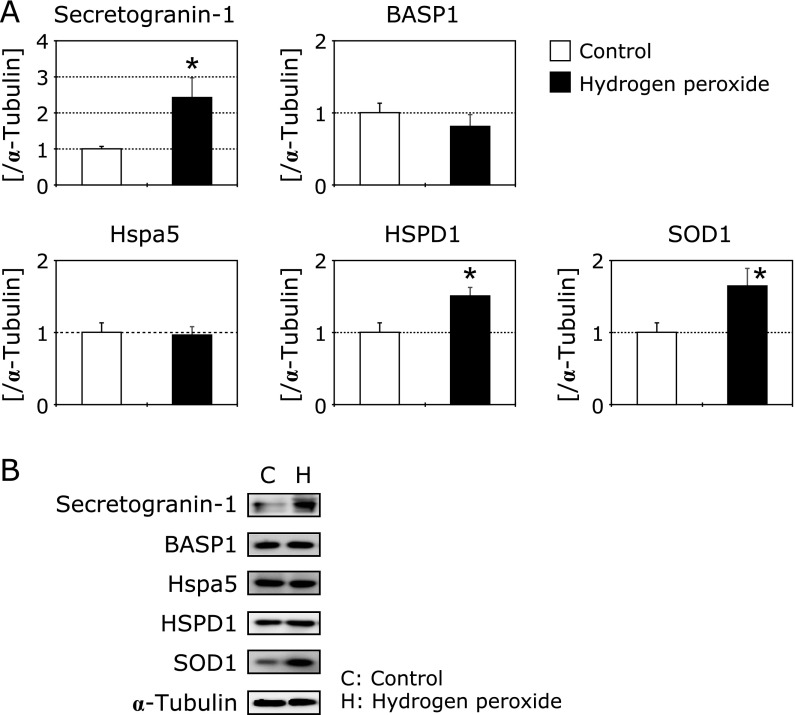
Changes in neurite-specific protein expressions in isolated neurites of hydrogen peroxide-treated N1E-115 cells (A). Black columns show hydrogen peroxide-treated cells (*n* = 4) and white columns show control cells (*n* = 4). The ratio of each protein band intensity to α-tubulin intensity are shown, with ratios of control samples set to 1. Each sample was collected from 12 inserts, and each column represents the mean of three independent experiments. Data were analyzed using Dunnett’s test, indicates *p*<0.05. Western blots of each protein (B).

To determine whether the identified proteins could be detected in isolated neurites, we checked protein expression by western blotting. As shown in Fig. 5A and B, secretogranin-1, 60 kDa heat shock protein (HSPD) 1 and superoxide dismutase 1 were significantly increased in isolated neurites of hydrogen peroxide-treated cells. Brain acid soluble protein 1 (BASP1) and 78 kDa glucose-regulated protein (Hspa5) were not increased by the treatment of hydrogen peroxide.

### Identified proteins were increased in aged and AD model mouse brains

**Fig. 6:**
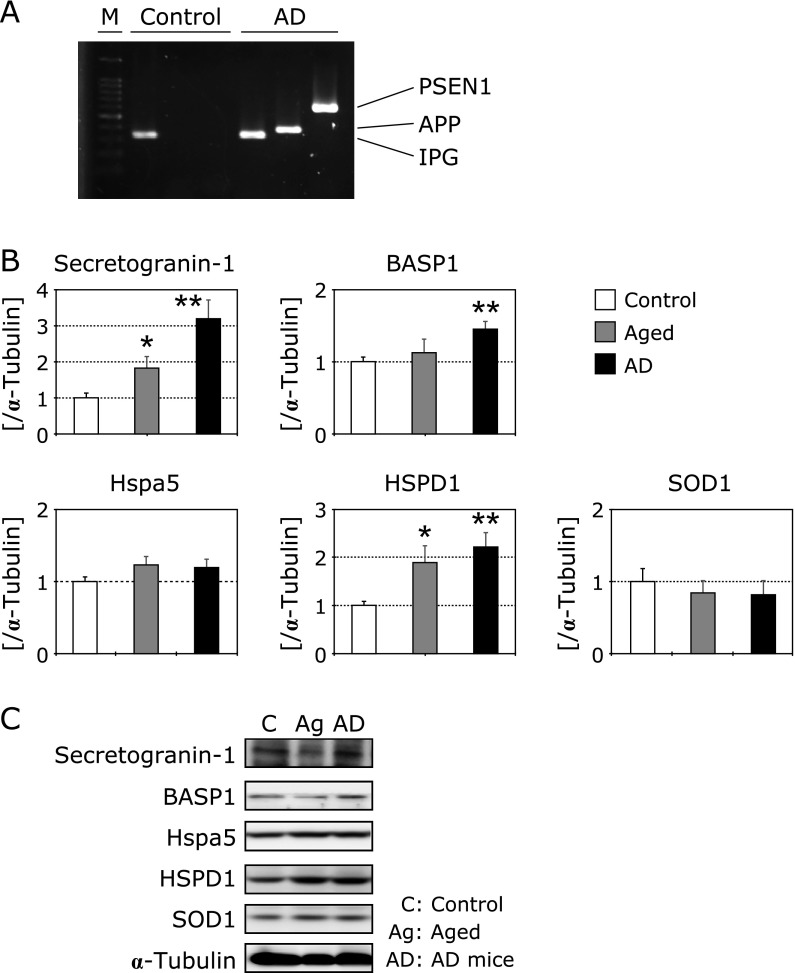
Genotyping of Alzheimer’s transgenic mice (A). Changes in proteins expressions in mice cerebral cortex (B). Black columns show 3-month-old Alzheimer’s transgenic mice (*n* = 9), and gray columns show aged mice (*n* = 9). White columns show 3-month-old control mice (*n* = 9). The ratios of each protein band intensity to α-tubulin intensity are shown, and the ratios of control samples were set to 1. Each column represents the mean of three independent experiments. Data were analyzed using Dunnett’s test, * indicates *p*<0.05 and ** indicates *p*<0.01. Western blot analysis of each protein (C).

To test the relationship between changes in the expressions of these proteins and aging or age-related neurodegenerative disorders, we used normal aged and AD transgenic mice and checked protein expression by western blotting. After genotyping, we separated normal and AD mice, and used as experimental animals (Fig. 6A). Secretogranin-1 and HSPD1 proteins were significantly increased in normal aged mice brains compared to the controls. Secretogranin-1, BASP1 and HSPD1 protein were significantly increased in the AD mice brains compared to the controls (Fig. 6B and C).

## Discussion

### Establishment of neurite isolation method in N1E-115 cells by a cell culture insert system

In the present study, we aimed to identify neurite-specific proteins in hydrogen peroxide-treated N1E-115 cells. We first needed to establish a new neurite isolation method. Because cell bodies and neurites were not separated in previous studies, including ours, it was not possible to determine what proteins are directly related to the induction of neurite degeneration.^(8,9,15)^ Certainly, many intracellular proteins are translated in the nucleus and transported to distal ends of neurites by the axonal transport system.^(16,17)^ However, neurites can expand and contract easily by external stimuli such as changes in pH, ionic composition, etc.^(18,19)^ Furthermore, we have found that treatment with a low concentrations of hydrogen peroxide induces neurite degeneration prior to cell death.^(8,9)^ It is well known that neurite degeneration starts from the distal end of neurites in *Wallerian Degeneration Slow* (*WLD*^*s*^) models.^(20)^ This indicates that neurite degeneration is an early sign of neuronal degeneration.

To determine changes in hydrogen peroxide-derived proteins expressions in neurite region of N1E-115 cells, we used a cell culture insert system to isolate neurites of N1E-115 cells. Cell culture insert systems are commonly used in cell to cell interaction experiments.^(21)^ There are many small pores in the insert of about 8 µm, and elongated neurites being found under the surface of the insert membrane (Fig. 2). To collect neurites, we scraped under the surface of inserts, and performed two different experiments to confirm the absence of cell bodies (Fig. 3). Both fluorescent staining and immunostaining showed no cell body regions at all, and so we used this method to isolate neurite samples for further experiments.

However, the total protein volume of isolated neurites was very low compared to whole cell lysates. In this study, a total of four 6-well plates were prepared per sample. Two plates were used for LC-MALDI-TOF/TOF analysis, and two for western blotting. To eliminate confounding variables as much as possible, we made two N1E-115 cell samples together in the presence or absence of hydrogen peroxide.

### Identification of proteins by LC-MALDI-TOF/TOF analysis in the presence or absence of hydrogen peroxide in isolated neurites of N1E-115 cells

First, we checked the reproducibility iTRAQ labeling. A control sample lysate was divided into two tubes equally, and each sample was labelled with different iTRAQ reagents (114 and 116). Each iTRAQ-labeled sample was mixed and analyzed two times by LC-MALDI-TOF/TOF. In this test analysis, we identified around 300 proteins. The iTRAQ ratio of identified proteins was roughly 0.8 to 1.3. The same 203 proteins were identified, and the matching ratio was to 57.5%. These results showed that our labelling method worked. Next, we studied the changes of protein profiles by the treatment with hydrogen peroxide in triplicate. When there was a difference more than 1.5 or less than 0.6 times, we defined as differentially expressed proteins. After LC-MALDI-TOF/TOF analysis, only proteins with an increased expression ratio by hydrogen peroxide treatment were selected (Fig. 4B). Furthermore, we excluded nuclear proteins, such as histones and ribonucleoprotein. Why nucleus-related proteins were detected in isolated nerites samples was unknown. It may be technical problem including contamination of cytoplasm. Finally, we selected 5 proteins and conducted further detailed studies by western blotting. On the other hand, the number of proteins with a decreased expression ratio by hydrogen peroxide treatment were 9 (data not shown). Large number of proteins were related to cytoskeletal proteins. Furthermore, we tried to analyze the levels of protein modifications such as phosphorylation, acetylation, polymerization, etc. However, this proved difficult. We were able to identify proteins and those that increased with hydrogen peroxide treatment. Detecting novel proteins and protein modifications are issues of current research.

### Secretogranin-1 increased in isolated neurites of hydrogen peroxide-treated N1E-115 cells, and in normal aged and AD mice brains

To determine the reproducibility of the 5 identified proteins, we checked their expressions by western blotting. The secretogranin-1 expression in isolated neurites of hydrogen peroxide-treated cells was significantly higher than that of the controls (Fig. 5). Secretogranin-1, also called chromogranin B, is an acidic secretory soluble protein and is expressed in neuroendocrine and neuronal cells.^(22)^ Secretogranin-1 belongs to a chromogranin family, which plays a crucial role in the sorting and aggregation of secretory products in the trans-Golgi network,^(23)^ and some researchers have used it as a marker of large dense core vesicles.^(24)^ Specifically, secretogranin-1 is highly expressed in excitatory hippocampal CA3 and DG neurons.^(25)^ Recently, it has been reported that a relationship exists between changes in the secretogranin-1 and the progression of some severe diseases. Monaghan *et al.*^(26)^ reported that the plasma secretogranin-1 level was increased in human gastroenteropancreatic neuroendocrine tumors. Secretogranin-1 has been identified in biopsy specimens of medullary thyroid carcinoma patients by LC-MS/MS.^(27)^ Kroksveen *et al.*^(28)^ reported that secretogranin-1 is increased in the cerebrospinal fluid of early multiple sclerosis patients, and may be used as a biomarker of this disease. Furthermore, several lines of evidence demonstrate a relationship between secretogranin-1 and AD. Chromogranin peptides including secretogranin-1 are located in neuritic plaques of the cortical layer in AD patients.^(24)^ The localization of secretogranin-1 is matched with amyloid plaques in AD transgenic mice brains.^(29)^ Mattsson *et al.*^(30)^ reported that the secretogranin-1 concentration is significantly decreased in early AD patients, and is one possible reason for the reduction of synapses. In our study, the expression of secretogranin-1 was significantly increased in AD transgenic mice brains compared to age-matched controls (Fig. 6). It is well known that the development and progression of AD is strongly correlated with ROS production and ROS-based neuronal injury.^(1,31,32)^ It is possibility that changes in secretogranin-1 expression in neurites relates to these neurodegenerative disorders in some form. However, further experiments are needed to elucidate the relationship between changes in ROS-related secretogranin-1 expression and AD progression. Additionally, the expression of secretogranin-1 was significantly increased in normal aged mice brains. Considering that secretogranin-1 expression was significantly increased in hydrogen peroxide-treated N1E-115 cells compared to untreated ones, our results may support the free radical theory of aging.

### HSPD1 is increased in isolated neurites of hydrogen peroxide-treated N1E-115 cells, and normal aged and AD mice brains

HSPD1 protein expression in isolated neurites of hydrogen peroxide-treated neurons was significantly increased compared to untreated ones (Fig. 5). Furthermore, HSPD1 was significantly increased in normal aged and AD transgenic mice brains compared to young controls (Fig. 6). HSPD1 is also known as HSP60, and is localized in the mitochondrial matrix space.^(33)^ In general, HSPs are upregulated in response to hyperthermia, an imbalance of the redox system, chemical stimulation from extracellular regions, etc.^(34)^ Much of the literature on HSP60 is related to immune diseases.^(35)^ However, several studies have demonstrated a relationship between changes in HSP60 and neurodegenerative disorders.^(34,36,37)^ HSP60 binds to amyloid precursor protein and beta-amyloid, and accumulates in the mitochondria of AD transgenic mice brains.^(34)^ HSP60 supports the folding of matrix proteins and plays a crucial role in protection against Parkinson’s disease.^(37)^ Additionally, in endothelial cells, the expression of HSP60 is a marker of chemical stress such as from smoking or free radicals, and increases the risk of atherosclerosis.^(35)^ In contrast to our results, Kleinridders *et al.*^(38)^ reported that mitochondrial dysfunction is induced by downregulation of HSP60 in diabetic mice, and ROS production and insulin resistance are increased. In our previous study, we found that mitochondria accumulate in neurite beading regions^(18)^ and induce superoxide production in hydrogen peroxide-treated neuronal cells.^(39)^ Mitochondria play crucial roles in the branching and elongation of neurites.^(40,41)^ It is possible that changes in mitochondrial function, including in HSP60, contribute to the maintenance of neurite function. We are continuing to study the relationship between changes in mitochondrial function and the induction of neurite degeneration, especially regarding the expression of ROS-related proteins.

In this study, we isolated neurites using a cell culture insert system and identified proteins increased in isolated neurites of hydrogen peroxide-treated N1E-115 cells. In particular, the expressions of secretogranin-1 and HSPD1 were significantly increased not only in isolated neurites with hydrogen peroxide, but also in normal aged and AD transgenic mice brains. More study is needed to elucidate the relationship between these proteins and ROS-related neurite degeneration, such as an overexpression and a knockdown techniques. These proteins and others may serve as new biomarkers prior to the induction of neurite degeneration caused by ROS.

## Abbreviations

AD: Alzheimer’s disease
BASP1: brain acid soluble protein 1
BSA: bovine serum albumin
CAT: catalase
CRMP: collapsin response mediator protein
DMSO: dimethyl sulfoxide
FCS: fetal calf serum
GPx: glutathione peroxidase
HSP: heat shock protein
Hspa5: heat shock 70-kDa protein 5
HSPD1: heat shock protein family D (Hsp60) member 1
IL: interleukin
LC-MALDI-TOF/TOF: liquid chromatography-matrix-assisted laser desorption/ionization-time of flight/time of flight
MAP-LC3: microtubule associated protein-light chain 3
PBS: phosphate-buffered saline
PFA: paraformaldehyde
ROS: reactive oxygen species
RT: room temperature
SOD: superoxide dismutase
TgAPP: transgenic amyloid precursor protein
TgPSEN1: transgenic presenilin 1
*WLD*^*s*^: *Wallerian Degeneration Slow*
