# Supplementary material for: Proteomic study on neurite responses to oxidative stress: search for differentially expressed proteins in isolated neurites of N1E-115 cells
Source: J Clin Biochem Nutr. 2018 Aug 11;64(1):36–44. doi: 10.3164/jcbn.18-31 (PMC6348415; doi:10.3164/jcbn.18-31)
Supplement: Supplemental Table 1 [file jcbn18-31st01.pdf]

Supplemental Table 1. The result of LC-MALDI-TOF/TOF analysis

Unused ≥1.3 Peptides (95%) ≥1, iTRAQ114 and iTRAQ117		*Yellow boxes: Peptides = 1								
Red: more than 1.5 times difference,<br>Green: less than 0.67 times difference		Unused			Peptides			H_2O_2/Control		
Accession No.	NAME	RUN1	RUN2	RUN3	RUN1	RUN2	RUN3	RUN1	RUN2	RUN3
Q64433	10 kDa heat shock protein, mitochondrial	13.59	11.68	9.11	11	6	5	1.02	1.04	1.06
Q9CQV8	14-3-3 protein beta/alpha	2.04	2	2	4	3	3	1.21	1.20	0.99
P61982	14-3-3 protein gamma	5.46	5.53	4.62	4	4	4	0.99	0.86	0.83
P68254	14-3-3 protein theta	2.27	2.52	4	3	3	3	1.11	0.99	0.99
P63101	14-3-3 protein zeta/delta	7.64	7.05	10	7	7	5	0.95	0.90	0.78
Q924T2	28S ribosomal protein S2, mitochondrial		2	2		1	1		1.69	1.17
Q80ZS3	28S ribosomal protein S26, mitochondrial	1.48			1			0.92		
Q9CQX8	28S ribosomal protein S36, mitochondrial	2.94	2		2	1		0.99	1.16	
O35972	39S ribosomal protein L23, mitochondrial		1.85			1			1.02	
Q921H8	3-ketoacyl-CoA thiolase A, peroxisomal	2		2	1		1	1.03		1.58
Q99J99	3-mercaptopyruvate sulfurtransferase	3.6	1.62		2	1		0.96	0.99	
P63325	40S ribosomal protein S10	12.16	8.49	4.45	7	5	2	1.07	1.06	2.03
P62301	40S ribosomal protein S13	2.79	2.33	2.11	1	1	1	1.07	0.88	0.97
P62264	40S ribosomal protein S14	6.78	1.4	3.27	3	2	2	0.97	0.86	1.05
P14131	40S ribosomal protein S16	2.08	3.19	3.05	1	2	2	0.94	0.96	0.99
P62270	40S ribosomal protein S18		2.77			1			0.86	
Q9CZX8	40S ribosomal protein S19	6.74	7.17	5.41	5	5	2	0.98	0.86	1.02
P60867	40S ribosomal protein S20	4	6	3.95	3	3	2	0.87	0.99	0.95
P62852	40S ribosomal protein S25	2.34		2.17	1		1	0.97		0.99
Q6ZWU9	40S ribosomal protein S27	1.71			2			0.96		
P62274	40S ribosomal protein S29		2.51			1			1.04	
P62862	40S ribosomal protein S30	2			1			1.12		
P97351	40S ribosomal protein S3a	2	3.13		1	2		0.96	0.96	
P62082	40S ribosomal protein S7			1.56			1			1.13
P14206	40S ribosomal protein SA	2	2.17	2	1	1	1	0.93	0.99	1.15
P56379	6.8 kDa mitochondrial proteolipid	2			1			1.08		
P63038	60 kDa heat shock protein, mitochondrial	24.18	25.22	27.65	20	15	16	1.00	1.00	2.00
P47963	60S ribosomal protein L13		1.37			1			0.99	
P67984	60S ribosomal protein L22			2			1			1.00
P61255	60S ribosomal protein L26	2	2.82	2	1	2	1	0.82	0.99	0.99
P47915	60S ribosomal protein L29		1.92	3.04		1	2		0.96	0.99
P62900	60S ribosomal protein L31	4	4.08	5.57	2	2	3	0.88	0.99	1.11
P47911	60S ribosomal protein L6	1.51	2.12	2.15	1	1	2	0.99	0.99	0.99
P14148	60S ribosomal protein L7	1.52			1			0.79		
P12970	60S ribosomal protein L7a			2.9			2			1.08
P20029	78 kDa glucose-regulated protein	20.07	10.52	19.04	12	5	9	1.00	1.00	2.31
Q99KI0	Aconitate hydratase, mitochondrial	4.33	5.76	5.46	4	4	3	1.03	1.00	0.98
P60710	Actin, cytoplasmic 1			28.11			21			0.99
P63260	Actin, cytoplasmic 2	27.02	23.78		21	14		0.97	0.97	
O55137	Acyl-coenzyme A thioesterase 1		2			1			0.99	
Q9QYR9	Acyl-coenzyme A thioesterase 2, mitochondrial	2			1			1.02		
Q9WTP6	Adenylate kinase 2, mitochondrial	4.28			2			0.95		
P09242	Alkaline phosphatase, tissue-nonspecific isozyme		2			1			1.01	
Q7TPR4	Alpha-actinin-1	2	7.2		3	3		0.88	1.08	
P57780	Alpha-actinin-4	8.02	2	4.01	5	2	2	1.03	0.99	0.97
P17182	Alpha-enolase	10.29	5.28	8.6	5	3	4	0.96	0.96	0.92
P46660	Alpha-internexin	9.02	1.51	1.35	6	2	1	0.99	0.91	0.97
P07356	Annexin A2	2	2	2	1	1	1	0.99	0.99	0.90
P97429	Annexin A4	2	1.8	2.46	1	1	1	0.79	0.74	0.99
P14824	Annexin A6	2	1.92		1	1		1.21	0.90	
P05202	Aspartate aminotransferase, mitochondrial	3.59	3.66	2.03	2	2	1	1.04	1.09	1.02
P03930	ATP synthase protein 8	2			1			0.73		
Q03265	ATP synthase subunit alpha, mitochondrial	18.86	15.41	15.93	14	12	9	0.97	0.97	0.99
Q9CQQ7	ATP synthase subunit b, mitochondrial	2		2.74	1		1	0.92		0.99
P56480	ATP synthase subunit beta, mitochondrial	28.67	24.22	19.01	20	13	11	0.98	0.97	0.72
Q9DCX2	ATP synthase subunit d, mitochondrial	9.21	7.41	4.88	7	4	3	1.02	1.02	0.98

Unused ≥1.3 Peptides (95%) ≥1, iTRAQ114 and iTRAQ117		*Yellow boxes: Peptides = 1								
Red: more than 1.5 times difference,<br>Green: less than 0.67 times difference		Unused			Peptides			H_2O_2/Control		
Accession No.	NAME	RUN1	RUN2	RUN3	RUN1	RUN2	RUN3	RUN1	RUN2	RUN3
Q9D3D9	ATP synthase subunit delta, mitochondrial	8.29	6.09	6.1	5	4	4	1.00	0.99	1.01
Q06185	ATP synthase subunit e, mitochondrial	2.04			1			1.16		
Q9DB20	ATP synthase subunit o, mitochondrial	6	2.22	2.93	3	1	2	0.97	0.94	0.70
P97450	ATP synthase-coupling factor 6, mitochondrial		1.82			1			1.17	
O35143	ATPase inhibitor, mitochondrial	7.12		1.58	4		1	0.99		1.04
P18572	Basigin	4	5.12	6.07	2	3	3	0.99	0.99	1.01
P59017	Bcl-2-like protein 13	1.59			1			1.12		
Q91XV3	Brain acid soluble protein 1	13.51	8.03	10	12	7	7	1.38	1.00	2.70
Q9CXW3	Calcyclin-binding protein	2.24		1.9	1		1	1.14		1.08
P35564	Calnexin		2			1			1.09	
P14211	Calreticulin	2.01			1			0.95		
O35887	Calumenin	2.19	2	2	1	1	1	0.97	1.07	1.13
Q9DBC7	cAMP-dependent protein kinase type I-alpha regulatory subunit		2			1			1.01	
Q80ZM8	Cardiolipin synthase			2			1			1.16
Q91WS0	CDGSH iron sulfur domain-containing protein 1	2		2	1		1	0.96		1.11
Q9JKC6	Cell cycle exit and neuronal differentiation protein 1		2	2.52		1	1		0.66	1.29
Q6A065	Centrosomal protein of 170 kDa	2			1			1.07		
P26339	Chromogranin-A		1.47			1			1.54	
Q9CZU6	Citrate synthase, mitochondrial	2.01	4.1	4.04	1	2	2	0.83	0.99	0.95
Q9CQI6	Coactosin-like protein	1.46			1			0.97		
P18760	Cofilin-1	8.45	6.35	7.78	4	3	4	1.00	1.05	0.97
Q9D2R6	Coiled-coil domain-containing protein 56	4.02			2			1.03		
Q9D1L0	Coiled-coil-helix-coiled-coil-helix domain-containing protein 2, mitochondrial	2			1			0.83		
Q9CRB9	Coiled-coil-helix-coiled-coil-helix domain-containing protein 3, mitochondrial	12.27	7.34	5.95	7	4	3	1.01	1.06	0.98
Q91VN4	Coiled-coil-helix-coiled-coil-helix domain-containing protein 6	2.13			1			0.99		
P11087	Collagen alpha-1(I) chain		7.3	28.79		15	14		0.52	0.44
Q01149	Collagen alpha-2(I) chain		9.68	23.82		14	15		0.12	0.20
O35658	Complement component 1 Q subcomponent-binding protein, mitochondrial	2.78	3.02		4	3		1.01	1.18	
Q8VBV7	COP9 signalosome complex subunit 8	2			1			0.66		
O08997	Copper transport protein ATOX1	2			1			1.12		
Q9CQX2	Cytochrome b5 type B			4			2			0.99
Q9CZ13	Cytochrome b-c1 complex subunit 1, mitochondrial	3.8	2.24	2.31	2	1	1	1.00	0.93	0.97
Q9CQ69	Cytochrome b-c1 complex subunit 8	4.9	3.82	4.37	3	2	3	0.77	0.99	0.95
Q9CR68	Cytochrome b-c1 complex subunit Rieske, mitochondrial	4			2			1.02		
P56394	Cytochrome c oxidase copper chaperone	2.86	1.88	2.51	3	1	1	1.01	0.98	0.85
P19783	Cytochrome c oxidase subunit 4 isoform 1, mitochondrial	4.49	3.18	2.66	5	2	2	1.07	1.10	1.13
Q91W29	Cytochrome c oxidase subunit 4 isoform 2, mitochondrial	1.7			1			0.92		
P19536	Cytochrome c oxidase subunit 5B, mitochondrial	4.77	6	4.74	4	4	4	1.00	0.99	1.14
P56391	Cytochrome c oxidase subunit 6B1	2.13			1			1.14		
Q9CPQ1	Cytochrome c oxidase subunit 6C	8.33	7.38	10.65	6	3	5	1.04	0.98	1.03
P62897	Cytochrome c, somatic	2.72	2.24	2.05	3	1	1	1.13	1.05	0.90
Q9D0M3	Cytochrome c1, heme protein, mitochondrial	3.26	2.42	2.3	2	1	1	0.95	0.98	0.91
O88487	Cytoplasmic dynein 1 intermediate chain 2	2			1			0.70		
Q80WW9	DDRGK domain-containing protein 1		1.44			1			1.04	
Q6P253	Dermokine	2.42		2	2		1	0.96		1.08
O08749	Dihydrolipoyl dehydrogenase, mitochondrial		2			1			0.90	
Q8BMF4	Dihydrolipoyllysine-residue acetyltransferase component of pyruvate dehydrogenase complex, mitochondrial	2.31	3.05	2.05	1	3	1	0.99	0.96	0.82

Unused ≥1.3 Peptides (95%) ≥1, iTRAQ114 and iTRAQ117		*Yellow boxes: Peptides = 1								
Red: more than 1.5 times difference,<br>Green: less than 0.67 times difference		Unused			Peptides			H_2O_2/Control		
Accession No.	NAME	RUN1	RUN2	RUN3	RUN1	RUN2	RUN3	RUN1	RUN2	RUN3
Q9D2G2	Dihydrolipoyllysine-residue succinyltransferase component of 2-oxoglutarate dehydrogenase complex, mitochondrial	5.17	4.92	5.07	3	4	3	0.96	0.98	0.96
P97427	Dihydropyrimidinase-related protein 1	2			4			1.20		
O08553	Dihydropyrimidinase-related protein 2	19.51	14.57	11.85	12	8	5	0.97	0.87	0.83
Q62188	Dihydropyrimidinase-related protein 3	8	4.84	4.3	7	4	2	0.97	0.80	0.99
Q8K1M6	Dynamin-1-like protein	2			1			1.16		
Q9DCW4	Electron transfer flavoprotein subunit beta	2.35			1			0.99		
Q921G7	Electron transfer flavoprotein-ubiquinone oxidoreductase, mitochondrial	1.79			1			0.82		
P10126	Elongation factor 1-alpha 1	13.63	12.47	8.69	8	8	5	0.99	1.01	0.97
P57776	Elongation factor 1-delta	5.44			3			0.96		
Q8BFR5	Elongation factor Tu, mitochondrial		4			2			1.27	
O08579	Emerin	2			1			1.02		
Q62419	Endophilin-A2		1.85			1			0.82	
Q8R3V5	Endophilin-B2	1.89			1			1.33		
P08113	Endoplasmin	3.29	4.96		2	4		0.89	0.99	
P54116	Erythrocyte band 7 integral membrane protein	2.24		1.55	1		1	1.12		0.87
P60843	Eukaryotic initiation factor 4A-I	2	1.92	2	1	1	1	0.90	0.79	0.76
Q8JZQ9	Eukaryotic translation initiation factor 3 subunit B	4			2			1.04		
Q80XI3	Eukaryotic translation initiation factor 4 gamma 3	1.41			1			0.69		
Q8BGD9	Eukaryotic translation initiation factor 4B	5.34			4			0.97		
Q9WUK2	Eukaryotic translation initiation factor 4H	1.54			2			0.97		
P29391	Ferritin light chain 1	2.59	4.2	2.74	1	3	1	1.05	0.95	0.94
P05064	Fructose-bisphosphate aldolase A	12.22	7.09	4.14	7	4	2	1.01	0.97	0.99
P16858	Glyceraldehyde-3-phosphate dehydrogenase	2.49	4.74	4.44	1	3	2	0.98	1.01	0.95
Q05915	GTP cyclohydrolase 1	2.39			2			0.98		
P01900	H-2 class I histocompatibility antigen, D-D alpha chain	1.64			1			0.99		
P63017	Heat shock cognate 71 kDa protein	10.69	8.89	10.01	8	7	5	1.00	0.99	0.96
P11499	Heat shock protein HSP 90-beta	2.17			1			0.91		
Q9R257	Heme-binding protein 1	3.48			2			1.00		
P01942	Hemoglobin subunit alpha	4.25	2.31	3.29	4	2	2	1.07	1.14	1.00
Q99020	Heterogeneous nuclear ribonucleoprotein A/B	4		2.07	2		1	1.18		1.56
P49312	Heterogeneous nuclear ribonucleoprotein A1	7.6	4.48	5.83	5	4	4	1.05	1.34	9.55
P61979	Heterogeneous nuclear ribonucleoprotein K	4.42		3.65	2		3	1.00		2.31
Q8R081	Heterogeneous nuclear ribonucleoprotein L			1.3			1			2.00
Q9D0E1	Heterogeneous nuclear ribonucleoprotein M	1.38			1			0.99		
Q8VEK3	Heterogeneous nuclear ribonucleoprotein U	2.6	2.02	5.6	1	1	3	0.95	1.17	1.06
O88569	Heterogeneous nuclear ribonucleoproteins A2/B1		5.45			4			1.05	
P70349	Histidine triad nucleotide-binding protein 1	2			1			1.06		
Q9D0S9	Histidine triad nucleotide-binding protein 2, mitochondrial	1.3			1			0.83		
P10922	Histone H1.0	1.66			1			1.08		
P15864	Histone H1.2			7.71			4			3.05
Q8CGP6	Histone H2A type 1-H	10.36			6			0.99		
Q6GSS7	Histone H2A type 2-A			9.36			3			2.09
Q64523	Histone H2A type 2-C			1.42			3			3.02
Q8BFU2	Histone H2A type 3		5.55			4			1.05	
P27661	Histone H2A.X			4.42			4			4.29
P0C0S6	Histone H2A.Z			5.72			5			3.02
Q6ZWY9	Histone H2B type 1-C/E/G	23.65	18.54		14	11		0.98	1.82	
P10853	Histone H2B type 1-F/J/L			27.06			15			1.05
P84228	Histone H3.2	6.28		12.25	4		6	0.92		5.11
P84244	Histone H3.3		7.62			3			1.00	
P62806	Histone H4	6.11	4.59	9.04	4	2	5	0.95	0.96	12.47
P03975	IgE-binding protein	9.8	12.17	13.92	6	8	10	0.99	1.04	2.86

Unused ≥1.3 Peptides (95%) ≥1, iTRAQ114 and iTRAQ117		*Yellow boxes: Peptides = 1								
Red: more than 1.5 times difference,<br>Green: less than 0.67 times difference		Unused			Peptides			H_2O_2/Control		
Accession No.	NAME	RUN1	RUN2	RUN3	RUN1	RUN2	RUN3	RUN1	RUN2	RUN3
Q9D819	Inorganic pyrophosphatase	2			1			1.01		
Q9DCB8	Iron-sulfur cluster assembly 2 homolog, mitochondrial	2			1			1.12		
Q9D6R2	Isocitrate dehydrogenase [NAD] subunit alpha, mitochondrial	4	1.7	4.08	2	1	2	0.83	0.98	0.99
Q6P253-2	Isoform 2 of Dermokine		1.38			1			1.24	
Q8BFR5-2	Isoform 2 of Elongation factor Tu, mitochondrial	4		3.72	2		2	1.01		1.15
Q8BG05-2	Isoform 2 of Heterogeneous nuclear ribonucleoprotein A3	2.35	2	4	3	1	3	1.25	0.99	1.66
Q60715-2	Isoform 2 of Prolyl 4-hydroxylase subunit alpha-1	2.22			1			1.01		
Q64337-2	Isoform 2 of Sequestosome-1	2	1.66		1	1		1.08	1.08	
Q9CZY3-2	Isoform 2 of Ubiquitin-conjugating enzyme E2 variant 1	3.15			2			0.99		
Q62418-3	Isoform 3 of Drebrin-like protein	2.4			1			0.90		
P57776-3	Isoform 3 of Elongation factor 1-delta		2			1			1.37	
O88569-3	Isoform 3 of Heterogeneous nuclear ribonucleoproteins A2/B1			8.71			5			6.92
Q8CAQ8-5	Isoform 5 of Mitochondrial inner membrane protein	12.74			7			0.96		
P15331-3	Isoform 5b of Peripherin	1.36	25.62	24.04	29	16	18	0.96	1.02	0.96
P04370-9	Isoform 9 of Myelin basic protein		3.16			2			0.66	
O88569-2	Isoform A2 of Heterogeneous nuclear ribonucleoproteins A2/B1	16.41			9			1.02		
P21107-2	Isoform Cytoskeletal of Tropomyosin alpha-3 chain	6.18	3.6	2.34	4	3	2	0.99	1.02	1.00
Q60716-2	Isoform IIa of Prolyl 4-hydroxylase subunit alpha-2		3.5			2			1.03	
Q3TTY5	Keratin, type II cytoskeletal 2 epidermal	2			1			1.01		
Q61768	Kinesin-1 heavy chain	2	1.52		1	1		1.08	0.86	
P48678	Lamin-A/C	2.16		2	1		1	1.05		2.31
Q61033	Lamina-associated polypeptide 2, isoforms alpha/zeta	2			1			1.18		
Q61792	LIM and SH3 domain protein 1	2			1			1.00		
Q8CGK3	Lon protease homolog, mitochondrial	2	2.14	2	1	1	1	1.41	0.95	1.00
P08249	Malate dehydrogenase, mitochondrial	4.43	2.88	11.59	3	1	5	1.02	0.97	1.04
P28667	MARCKS-related protein	2.02	2.03	2	1	1	1	0.97	1.03	1.02
Q8BI84	Melanoma inhibitory activity protein 3	2.02			1			0.81		
O55022	Membrane-associated progesterone receptor component 1	3.51	2.87		2	2		1.01	1.02	
P14873	Microtubule-associated protein 1B	24.3	8.97	7.68	13	4	4	1.00	0.94	1.00
Q7TSJ2	Microtubule-associated protein 6		2.63	2.63		2	1		0.58	0.99
P10637	Microtubule-associated protein tau	4.03	2		2	1		1.18	1.18	
Q6PCP5	Mitochondrial fission factor	4.16		2.02	2		1	1.01		0.75
Q9WV98	Mitochondrial import inner membrane translocase subunit Tim9	2			1			1.01		
Q8CAQ8	Mitochondrial inner membrane protein		6.6	7.49		3	4		1.02	0.99
P63085	Mitogen-activated protein kinase 1	1.48		1.57	1		1	0.84		0.90
P26041	Moesin	2.35			2			0.82		
P53986	Monocarboxylate transporter 1		2	2		1	1		1.31	1.25
P57787	Monocarboxylate transporter 4	2			1					
Q3V3R1	Monofunctional C1-tetrahydrofolate synthase, mitochondrial	2			1			0.95		
O08539	Myc box-dependent-interacting protein 1	2			1			0.99		
Q60605	Myosin light polypeptide 6	2	2.44	2.8	1	1	1	1.50	1.21	0.99
Q3THE2	Myosin regulatory light chain 12B	2	4	2	1	2	1	0.97	0.99	1.01
Q61879	Myosin-10		4.31	2		4	2		0.88	0.99
Q8VDD5	Myosin-9	11.22	10.17	12.8	5	5	6	1.02	0.99	1.00
Q8BFR4	N-acetylglucosamine-6-sulfatase	1.82			1			0.97		
Q9CQ75	NADH dehydrogenase [ubiquinone] 1 alpha subcomplex subunit 2	4		2.57	2		1	1.06		1.04

Unused ≥1.3 Peptides (95%) ≥1, iTRAQ114 and iTRAQ117		*Yellow boxes: Peptides = 1								
Red: more than 1.5 times difference,<br>Green: less than 0.67 times difference		Unused			Peptides			H_2O_2/Control		
Accession No.	NAME	RUN1	RUN2	RUN3	RUN1	RUN2	RUN3	RUN1	RUN2	RUN3
O09111	NADH dehydrogenase [ubiquinone] 1 beta subcomplex subunit 11, mitochondrial	1.51			1			0.69		
Q9CQC7	NADH dehydrogenase [ubiquinone] 1 beta subcomplex subunit 4	1.44			1			0.94		
Q9DCT2	NADH dehydrogenase [ubiquinone] iron-sulfur protein 3, mitochondrial	2	2	2	1	1	1	0.89	0.99	1.08
Q99LY9	NADH dehydrogenase [ubiquinone] iron-sulfur protein 5	1.8			1			1.16		
P52503	NADH dehydrogenase [ubiquinone] iron-sulfur protein 6, mitochondrial	2			1			0.88		
P61082	NEDD8-conjugating enzyme Ubc12	3.3			2			0.99		
P60761	Neurogranin		2			1			0.51	
P06837	Neuromodulin	10.81	8.73	8.43	10	6	5	1.01	1.05	0.98
Q9QZ23	NFU1 iron-sulfur cluster scaffold homolog, mitochondrial	4.03	1.42	2.51	2	1	1	0.99	1.00	0.96
P09602	Non-histone chromosomal protein HMG-17			3.8			2			6.49
Q9CZ44	NSFL1 cofactor p47	11.78	2.31		6	1		0.99	1.09	
O35685	Nuclear migration protein nudC	2.05			1			1.00		
P09405	Nucleolin	11.21			6			0.56		
Q61937	Nucleophosmin	12.53	2.49	7.07	7	2	4	1.05	1.03	5.20
Q01768	Nucleoside diphosphate kinase B	3.25	1.54		2	1		1.02	1.49	
Q9D0J8	Parathymosin	1.62			1			1.27		
P17742	Peptidyl-prolyl cis-trans isomerase A	19.58	9.87	10.79	14	5	5	0.99	1.04	0.99
P26883	Peptidyl-prolyl cis-trans isomerase FKBP1A	4			2			0.98		
P15331	Peripherin	35.55			31			0.89		
P35700	Peroxiredoxin-1	4	2.72	3.04	2	2	2	1.03	1.10	0.90
Q61171	Peroxiredoxin-2	2			1			0.99		
Q9R0A0	Peroxisomal membrane protein PEX14	2			1			1.13		
Q6P8I4	PEST proteolytic signal-containing nuclear protein	2			1			0.91		
Q8VEM8	Phosphate carrier protein, mitochondrial	1.34	2	2	1	1	1	0.95	0.99	0.95
Q8BH04	Phosphoenolpyruvate carboxykinase [GTP], mitochondrial	4.6	3.5		2	2		0.95	0.97	
Q9D0F9	Phosphoglucomutase-1	2			1			1.03		
P09411	Phosphoglycerate kinase 1	4.38	2.55	3.32	4	3	2	1.03	0.99	0.86
Q9CY58	Plasminogen activator inhibitor 1 RNA-binding protein	6.19			4			1.02		
Q9QXS1	Plectin-1	58.32	33.35	24.71	27	17	12	1.00	1.00	0.96
Q61990	Poly(rC)-binding protein 2	2			1			0.97		
P29341	Polyadenylate-binding protein 1		1.38			1			1.16	
Q9CWM4	Prefoldin subunit 1	2	1.64		1	1		1.21	1.94	
O70591	Prefoldin subunit 2	2			1			1.11		
Q9R0E1	Procollagen-lysine,2-oxoglutarate 5-dioxygenase 3	2	1.66		1	1		0.90	0.99	
P67778	Prohibitin	9.27	11.01	7.34	5	6	4	0.95	1.02	1.00
O35129	Prohibitin-2	3.67	3.29	4.52	2	2	2	1.03	0.97	1.17
Q60716	Prolyl 4-hydroxylase subunit alpha-2	4		2	2		1	1.03		1.05
Q4VAA2	Protein CDV3	1.37			1			1.36		
P27773	Protein disulfide-isomerase A3	13.12	10	14.99	9	5	7	0.98	1.04	1.07
Q922R8	Protein disulfide-isomerase A6			1.46			1			0.91
P09103	Protein disulfide-isomerase	7.18	5.47	6.51	5	3	3	1.04	0.95	1.20
Q99LX0	Protein DJ-1	2.88			2			0.79		
Q9CR98	Protein FAM136A	6.05			3			0.95		
Q9D6U8	Protein FAM162A	3.32			2			1.02		
Q8JZS0	Protein lin-7 homolog A	2			1			0.98		
Q7TQ95	Protein lunapark	1.6			1			0.90		
Q80WJ7	Protein LYRIC	2		1.79	1		1	1.00		1.02
O55125	Protein NipSnap homolog 1	4.39	2.52	2.1	2	1	1	0.95	0.93	0.95
Q9DBR7	Protein phosphatase 1 regulatory subunit 12A	2.83			2			0.99		
Q8R404	Protein QIL1	2			1			1.06		

Unused ≥1.3 Peptides (95%) ≥1, iTRAQ114 and iTRAQ117		*Yellow boxes: Peptides = 1								
Red: more than 1.5 times difference, Green: less than 0.67 times difference		Unused			Peptides			H_2O_2/Control		
Accession No.	NAME	RUN1	RUN2	RUN3	RUN1	RUN2	RUN3	RUN1	RUN2	RUN3
P63054	Purkinje cell protein 4	2	2		1	1		0.99	0.99	
P35486	Pyruvate dehydrogenase E1 component subunit alpha, somatic form, mitochondrial	4.46	2.02	2.28	2	1	1	1.00	1.00	0.97
Q9D051	Pyruvate dehydrogenase E1 component subunit beta, mitochondrial	2			1			1.22		
P52480	Pyruvate kinase isozymes M1/M2	7.57	6.72	3.43	4	4	2	0.99	0.97	0.37
Q6A0D4	Raftlin	1.49			1			0.95		
P97379	Ras GTPase-activating protein-binding protein 2	1.62			1			0.89		
P62492	Ras-related protein Rab-11A	10.79	6.71	5.3	5	3	3	1.00	0.96	0.98
P62823	Ras-related protein Rab-3C	2			1			1.10		
Q99P72	Reticulon-4	4	3.46	4	2	2	2	1.00	0.89	0.85
P52760	Ribonuclease UK114	2	2		1	1		0.75	0.99	
Q99PL5	Ribosome-binding protein 1	1.32	1.68	6.84	2	1	4	1.19	1.39	2.23
Q9WTM5	RuvB-like 2	2.14			1			0.90		
P16014	Secretogranin-1	101.62	69.84	51.73	72	46	28	1.42	2.17	1.91
Q03517	Secretogranin-2	9.91	3.12	3.11	5	2	2	0.95	1.18	1.08
Q8K021	Secretory carrier-associated membrane protein 1	1.57	2		1	1		0.91	1.10	
O55131	Septin-7	2		1.74	1		1	0.95		0.77
Q80UG5	Septin-9	1.85			1			1.08		
Q91VW3	SH3 domain-binding glutamic acid-rich-like protein 3	5.7	3.55		4	2		0.99	1.27	
P55194	SH3 domain-binding protein 1	2			1			0.68		
Q8VDN2	Sodium/potassium-transporting ATPase subunit alpha-1	5.55	4.07	1.83	3	2	1	0.95	0.82	0.75
Q91ZR2	Sorting nexin-18	2			1			0.90		
O70492	Sorting nexin-3	1.36			1			0.90		
P16546	Spectrin alpha chain, brain		1.6			1			0.90	
Q62261	Spectrin beta chain, brain 1	7.85	4.67	2.41	4	3	1	0.96	1.00	0.99
Q64674	Spermidine synthase	1.66			1			0.99		
Q8VIJ6	Splicing factor, proline- and glutamine-rich	6.2	2	2.02	3	1	1	0.98	1.20	2.09
Q9D8T7	SRA stem-loop-interacting RNA-binding protein, mitochondrial	2.07		2	1		1	1.04		1.26
Q60598	Src substrate cortactin	4.18			2			0.97		
P54227	Stathmin	6.36	2	2	5	1	1	1.01	1.38	0.67
Q99JB2	Stomatin-like protein 2		1.52	1.92		1	1		1.04	0.87
P38647	Stress-70 protein, mitochondrial	20.42	15.97	15.87	17	12	12	0.97	0.97	0.98
P08228	Superoxide dismutase [Cu-Zn]	5.78	3.37	2.26	4	3	2	1.06	1.13	1.54
P60879	Synaptosomal-associated protein 25	1.67			1			1.03		
Q70IV5	Synemin	2.36			1			0.99		
P61264	Syntaxin-1B		2			1			0.99	
O08599	Syntaxin-binding protein 1	2.84	4	2	2	2	1	1.07	1.06	0.99
P80317	T-complex protein 1 subunit zeta	3.11	1.74	2.39	2	1	1	0.86	1.05	0.99
Q9ER58	Testican-2	1.68	1.35		1	1		1.12	1.03	
Q6ZWY8	Thymosin beta-10	4		1.48	8		1	1.05		1.21
Q9CZY2	Transcription elongation factor A protein-like 8	1.74			1			1.02		
O35295	Transcriptional activator protein Pur-beta	1.8			1			1.04		
Q9QUI0	Transforming protein RhoA		2.37			2			1.04	
Q9WVA4	Transgelin-2	7.66	6.07	3.48	4	3	2	0.99	1.11	0.96
P40142	Transketolase		1.33			1			0.64	
P17751	Triosephosphate isomerase	8.66	4	5.6	5	2	3	1.02	1.06	0.96
Q9DCG9	tRNA methyltransferase 112 homolog	2			1			0.95		
P68373	Tubulin alpha-1C chain	5.38	5.6	4	5	3	2	0.98	0.99	0.99
Q7TMM9	Tubulin beta-2A chain		2	2		5	5		0.53	0.43
Q9CWF2	Tubulin beta-2B chain		2	1.31		4	5		0.83	0.65
P68372	Tubulin beta-2C chain	4.02			8			0.99		
Q9ERD7	Tubulin beta-3 chain		11.16	9.97		5	6		0.99	0.87
Q9D6F9	Tubulin beta-4 chain		4.82	1.74		5	4		0.53	0.32

Unused ≥1.3 Peptides (95%) ≥1, iTRAQ114 and iTRAQ117		*Yellow boxes: Peptides = 1								
Red: more than 1.5 times difference,<br>Green: less than 0.67 times difference		Unused			Peptides			H_2O_2/Control		
Accession No.	NAME	RUN1	RUN2	RUN3	RUN1	RUN2	RUN3	RUN1	RUN2	RUN3
P99024	Tubulin beta-5 chain	10.71	1.6	2.91	9	5	6	0.94	0.91	0.69
P48428	Tubulin-specific chaperone A	2.82		1.38	2		1	0.99		0.96
Q8K1Z0	Ubiquinone biosynthesis protein COQ9, mitochondrial	2			1			1.11		
Q9R0P9	Ubiquitin carboxyl-terminal hydrolase isozyme L1	4.3	1.91	2.28	3	1	1	0.99	1.02	0.99
P62991	Ubiquitin	8.16	6.77	3.91	5	3	2	1.03	0.90	0.98
P61089	Ubiquitin-conjugating enzyme E2 N	2			3			0.90		
Q8VE95	UPF0598 protein C8orf82 homolog	2	1.89	1.62	1	1	1	0.70	0.95	1.01
P20152	Vimentin	46.44	37.22	37.32	33	28	24	0.99	1.02	1.02
Q60932	Voltage-dependent anion-selective channel protein 1	2.65		1.66	1		2	0.97		1.15
P62814	V-type proton ATPase subunit B, brain isoform	3.29			2			0.91		
P50518	V-type proton ATPase subunit E 1	2.22		2.79	1		2	0.99		0.94
Q9D1K2	V-type proton ATPase subunit F	1.33			1			0.89		
Q9CR51	V-type proton ATPase subunit G 1	3.35			2			1.09		
A6PWY4	WD repeat-containing protein 76		1.42			1			0.67	

Red score shows that more than 1.5 times difference compared to the control. Green score shows that less than 0.67 times difference compared to the control. Yellow box shows that peptized score was 1. Blank box shows undetected.
